# Efficient solar photocatalyst based on cobalt oxide/iron oxide composite nanofibers for the detoxification of organic pollutants

**DOI:** 10.1186/1556-276X-9-510

**Published:** 2014-09-18

**Authors:** Safi Asim Bin Asif, Sher Bahadar Khan, Abdullah M Asiri

**Affiliations:** 1 Chemistry Department, Faculty of Science, King Abdulaziz University, P.O. Box 80203, Jeddah 21589, Saudi Arabia; 2 Center of Excellence for Advanced Materials Research, King Abdulaziz University, P.O. Box 80203, Jeddah 21589, Saudi Arabia

**Keywords:** Co_3_O_4_/Fe_2_O_3_, Nanofiber, Acridine orange, Brilliant cresyl blue, Organic pollutant, Solar photocatalyst

## Abstract

A Co_3_O_4_/Fe_2_O_3_ composite nanofiber-based solar photocatalyst has been prepared, and its catalytic performance was evaluated by degrading acridine orange (AO) and brilliant cresyl blue (BCB) beneath solar light. The morphological and physiochemical structure of the synthesized solar photocatalyst was characterized by X-ray diffraction (XRD), field emission scanning electron microscopy (FESEM), X-ray photoelectron spectroscopy (XPS), and Fourier transform infrared spectroscopy (FTIR). FESEM indicates that the Co_3_O_4_/Fe_2_O_3_ composite has fiber-like nanostructures with an average diameter of approximately 20 nm. These nanofibers are made of aggregated nanoparticles having approximately 8.0 nm of average diameter. The optical properties were examined by UV-visible spectrophotometry, and the band gap of the solar photocatalyst was found to be 2.12 eV. The as-grown solar photocatalyst exhibited high catalytic degradation in a short time by applying to degrade AO and BCB. The pH had an effect on the catalytic performance of the as-grown solar photocatalyst, and it was found that the synthesized solar photocatalyst is more efficient at high pH. The kinetics study of both AO and BCB degradation indicates that the as-grown nanocatalyst would be a talented and efficient solar photocatalyst for the removal of hazardous and toxic organic materials.

## Background

Recent industrial progress not only has positive consequences on human life but also causes big environmental threats due to the continuous release of industrial pollutants [1-3]. These pollutants cause too many serious problems for human and aquatic life because they are carcinogenic at trace levels for aquatic and non-aquatic environment [4-8]. Therefore, environmental pollution due to different types of pollutants created a center of global attention for the detoxification of these pollutants. A large number of physical, chemical, and biological methods were developed for water treatment but gained less importance because of low efficiency, high cost, and time consumption [9,10].

Among various techniques, photocatalysis in the presence of heterogeneous photocatalysts is considered as a cheap, easy, and efficient method for the decomposition of risky pollutants to final non-toxic products [11-15]. However, this technique is mainly depending on the photocatalyst which is mostly utilized for photocatalysis. TiO_2_ is the most active catalyst among them used so far [15]. Similarly, ZnO has also proven itself as one of the most active photocatalysts [16]. However TiO_2_, ZnO, and other similar photocatalysts can only degrade organic pollutants under UV light due to their large band gap, and thus, activation of these photocatalysts can only be carried out under UV light irradiation [15,16]. Therefore, utilization of these photocatalysts at a broad scale will result in small photoelectronic transition efficiency since the ultraviolet light comprises only 4% to 5% of the solar spectrum [17]. Therefore, based on energy conservation and environmental pollution concerns, there is a need to develop visible light-driven photocatalysts with enhanced efficiency.

Doped and composite nanostructure metal oxides have been considered as interesting materials and have shown excellent properties in photocatalysis [1,18]. Doping of nanomaterials modifies their features and characteristics. Nanocomposites generally increase numerous properties of metal oxide to fulfill the growing demand for various applications [19,20]. Semiconductors are the potential host material for transition materials and have attracted great attention due to their outstanding performance and multidimensional applications. Doping and nanocomposition improve the surface area and reduce the size of the metal oxide nanostructure. Doping of nanomaterials also tunes the band gap energy and enhances the conductivity, electrical, mechanical, barrier, sensing, and solar photocatalytic properties [21,22].

Cobalt oxide (Co_3_O_4_) nanomaterials have shown much application in different sectors. They have been used in Li-ion battery, catalysis, and sensing applications. All these properties depend on the particle size of Co_3_O_4_[23,24]. Similarly, Fe_2_O_3_ has been used in sensing and photocatalysis. However, the main drawback related to this metal oxide is its photocatalysis in the presence of UV [25]. Since cobalt oxide has oxidation catalysis and Fe_2_O_3_ has photocatalysis properties, the composite of these two oxides would probably show some interesting visible photocatalytic properties.

Therefore, in this research, a solar photocatalyst based on Co_3_O_4_/Fe_2_O_3_ composite nanofibers has been synthesized by an eco-friendly process and characterized by field emission scanning electron microscopy (FESEM), X-ray diffraction (XRD), X-ray photoelectron spectroscopy (XPS), Fourier transform infrared spectroscopy (FTIR), and UV-visible spectrophotometry. The catalytic properties of the as-grown solar photocatalyst were studied by degrading acridine orange (AO) and brilliant cresyl blue (BCB) at different pH under solar light.

## Methods

### Materials

Analytical reagent grade ferric nitrate nonahydrate Fe(NO_3_)_3_ · 9H_2_O, cobaltous nitrate hexahydrate Co(NO_3_)_2_ · 6H_2_O, sodium hydroxide NaOH, 99% ethanol C_2_H_5_OH, and other all chemicals were purchased from Sigma Aldrich (St. Louis, MO, USA).

### Synthesis and mechanism of Co_3_O_4_/Fe_2_O_3_ composite nanofibers

The required amount for 0.1 M aqueous solutions of Fe(NO_3_)_3_ · 9H_2_O and 0.2 M of Co(NO_3_)_2_ · 6H_2_O was accurately weighed. Then, both the salts were completely dissolved together in 100 mL distilled water at ambient temperature, and the homogeneous solution's pH was adjusted up to 11.0 by dropwise addition of freshly prepared 0.2 M NaOH solution under constant and vigorous stirring. After that, the solution was heated at about 60°C to 70°C with constant stirring overnight. After heating, the solution was allowed to cool at ambient temperature and centrifuged to separate the precipitate at 2,000 rpm. The supernatant solution was discarded, and the precipitate was washed thrice with ethanol. The precipitate was dried in an oven at 50°C to 60°C, ground, and stored in a clean, dry, and inert plastic vial.

### Growth mechanism of Co_3_O_4_/Fe_2_O_3_ composite nanofibers

The growth mechanisms of the composite nanofibers can be elucidated on the basis of chemical reactions as follows:

(1) $$\mathrm{NaOH}\left(\mathrm{aq}\right)\to {\mathrm{Na}}^{+}\left(\mathrm{aq}\right)+\;{\mathrm{OH}}^{-}\left(\mathrm{aq}\right)$$

(2) $$\mathrm{Fe}{\left({\mathrm{NO}}_{3}\right)}_{3}\cdot 9H_{2}O\;+\;3\mathrm{NaOH}\;\left(\mathrm{aq}\right)\to \mathrm{Fe}{\left(\mathrm{OH}\right)}_{3}\left(\mathrm{aq}\right)\;+\;3{\mathrm{NaNO}}_{3}+\;9H_{2}O$$

(3) $$\mathrm{Co}{\left({\mathrm{NO}}_{3}\right)}_{2}\cdot 6H_{2}O\;+\;2\mathrm{NaOH}\;\left(\mathrm{aq}\right)\to \mathrm{Co}{\left(\mathrm{OH}\right)}_{2}\left(\mathrm{aq}\right)\;+\;2{\mathrm{NaNO}}_{3}+\;6H_{2}O$$

(4) $$3\mathrm{Co}{\left(\mathrm{OH}\right)}_{2}\left(\mathrm{aq}\right)\;+\;2\mathrm{Fe}{\left(\mathrm{OH}\right)}_{3}\left(\mathrm{aq}\right)\;+\;2\mathrm{NaOH}\;\left(\mathrm{aq}\right)\to {\mathrm{Co}}_{3}O_{4}\cdot \;{\mathrm{Fe}}_{2}O_{3}+\;7H_{2}O\;+\;2{\mathrm{Na}}^{+}$$

### Characterization

The surface morphology of the nanoparticles was examined utilizing a JEOL scanning electron microscope (JSM-7600F, Akishima-shi, Japan). XRD patterns were obtained with a computer-controlled PANalytical X'Pert Explorer diffractometer (Almelo, The Netherlands). FTIR spectra were recorded in the range of 400 to 4,000 cm^−1^ on a PerkinElmer (Spectrum 100) FTIR spectrometer (Waltham, MA, USA). The UV spectrum was recorded from 200 to 800 nm using a UV-visible spectrophotometer (UV-2960, Labomed, Inc., Los Angeles, CA, USA).

### Photocatalytic degradation of dye

The photocatalytic property of the composite nanofibers was evaluated by using toxic dyes AO and BCB under solar light, which is relatively stable in the absence of a nanomaterial. This photocatalytic degradation of fluorescent cationic dye was evaluated taking two different 100.0 mL, 1E10 − 4 M transparent solutions of dye in a large surface area vessel, e.g., beakers. These solutions were then adjusted to different pH, 7.0 and 10.0. Sodium hydroxide (0.2 M) was used to adjust the pH under vigorous and continuous stirring. Then, exactly weighed 0.1 g (up to 1 mg) of nanomaterial was added into both solutions, and the solutions were kept in the dark to provide equilibration time for physical adsorption phenomenon of the material onto the dye surface. These solutions were simultaneously irradiated by sun daylight with continuous stirring. All three experiments were performed under sunlight. After that, an aliquot of 4 to 5 mL were pipetted out from each solution after regular pattern of irradiation and centrifuged, and the absorbance of the transparent solution at a wavelength maximum of 483.0 nm was measured by using a spectrophotometer (Labomed, Inc.). The same experiment was also performed in the absence of a photocatalyst to find out the control decolorization of dye.

## Results and discussion

### Physiochemical characterization of Co_3_O_4_/Fe_2_O_3_ composite nanofibers

#### Morphology study (FESEM)

**Figure 1:**
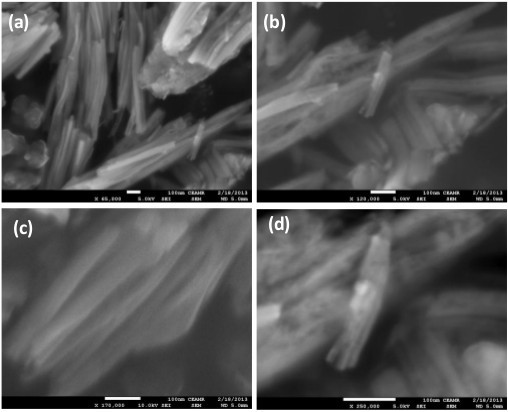
FESEM images of the composite nanofibers (a-d).

The morphology of the Co_3_O_4_/Fe_2_O_3_ nanocomposite was examined by FESEM and is shown in Figure 1. Low-magnification FESEM images displayed that the composite contains fiber-like morphology, and the average diameter of these fibers was determined to be approximately 20 nm. At high magnification, the FESEM indicates that the nanofibers are made of nanoparticles having approximately 8.0 nm of average diameter which aggregate and give a fiber-like nanostructure.

#### Phase and compositional study (XRD)

**Figure 2:**
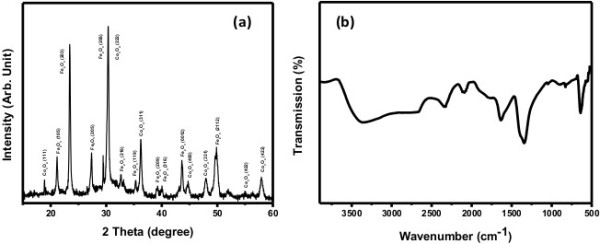
Powder XRD patterns (a) and FTIR spectrum (b) of the composite nanofibers.

The crystal phase and crystallinity of the composite nanofibers was checked by XRD which is shown in Figure 2a. XRD displayed sharp peaks for both Co_3_O_4_ and Fe_2_O_3_ which indicates that the synthesized product is a nanocomposite of cobalt and iron oxide. The peaks situated at 2*θ* = 18.9, 31.0, 36.5, 44.5, 48.2, 48.7, 55.2, and 58.8 are due to Co_3_O_4_ which are in good agreement with JCPDS (JCPDS # 80-1536) while all other peaks are attributed to Fe_2_O_3_ (JCPDS # 25-1402). The lattice parameters of Co_3_O_4_ are *a* = 8.1975 Å, *b* = 8.1975 Å, and *c* = 8.1975 Å with the *Fd3m* space group, and those of Fe_2_O_3_ are *a* = 8.3400 Å, *b* = 8.3400 Å, and *c* = 25.0200 Å with the *P* space group. The characteristic peaks of cubic Co_3_O_4_ are indexed to (111), (222), (311), (400), (331), (422), and (402) while the peaks of tetragonal Fe_2_O_3_ are indexed to (105), (203), (205), (206), (216), (119), (209), (316), (0012), and (2112). Thus, diffraction peaks suggests that the synthesized nanofibers are a composite of Co_3_O_4_ and Fe_2_O_3_.

#### FTIR analysis

The functional groups of the composite nanofibers were examined by FTIR as shown in Figure 2b. FTIR exhibited a peak for M-O-M stretching at 640 cm^−1^ along with some additional peaks for carbonate (1,341 cm^−1^) and water bending (1,632 cm^−1^) and stretching (3,361 cm^−1^). FTIR data suggests that the composite nanofibers are metal oxide-based nanostructures [2,3].

#### XPS

**Figure 3:**
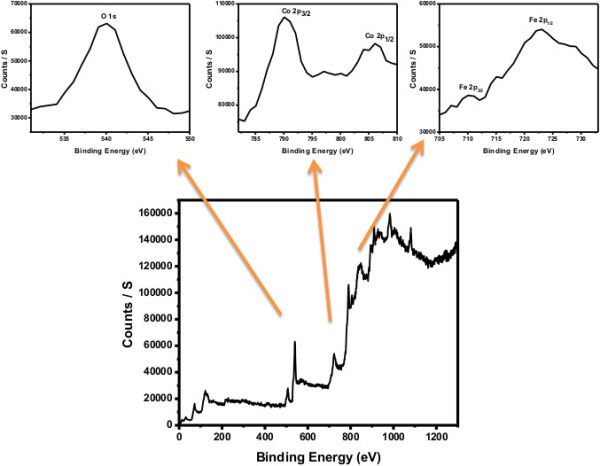
XPS spectrum of the composite nanofibers.

Figure 3 shows the XPS spectrum of the composite nanofibers which illustrates information about the bonding configuration and also explains the synthesized nanocomposite composition. The XPS spectrum of the composite nanofibers showed photoelectron peaks for O 1 s, Fe 2p_3/2_, Fe 2p_1/2_, Co 2p_3/2_, and Co 2p_1/2_ at binding energies of 539.8, 710.1, 722.8, 790.1, and 805.5 eV, correspondingly, which states that the composite nanofibers contain cobalt, iron, and oxygen. These results are in accordance with the reported values in literature for Co 2p_1/2_, Co 2p_3/2_, O 1 s, and Fe 2p_3/2_ peaks [24-26]. The XPS data reveal that that the as-grown composite nanofibers are made of Co_3_O_4_ and Fe_2_O_3_.

#### Photoabsorption properties and band gap energy

**Figure 4:**
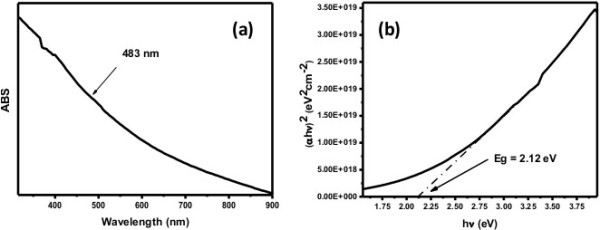
**UV-visible spectrum (a) and ( *αhν *)**^**2 **^**vs. *hν *** **plot (b) of the composite nanofibers.**

The optical property of the composite nanofibers was evaluated by UV-visible spectrophotometry. Since photocatalytic reaction is accounted for the electronic structure of the photocatalyst, there are two critical factors that influence photocatalysis: the amount of light absorbed by the material and the movement of light-induced electrons-holes. Theoretically, photoabsorption is proportional to the motion of electron-hole pairs, which in turn governs the probability of electron and hole towards reaction centers located at the surface of the photocatalyst. Figure 4a illustrates the strong absorption for the visible electromagnetic radiation which is credited to valence-conduction band transition (i.e., charge separation) [20]. The band gap energy *E*_*g*_ of the composite nanofibers is found to be 2.12 eV from the tangent displayed at the linear plateau of curve (*αhν*)^2^ vs. *hν* (Figure 4b).

### Photocatalytic activities

#### Effect of pH

**Figure 5:**
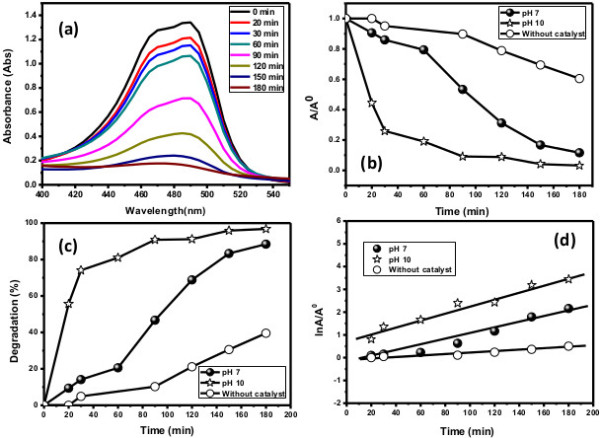
**Typical plots. (a)** Change in the absorption spectrum. **(b)** Comparison of change in absorbance vs. irradiation time. **(c)** Comparison of percentage of degradation vs. irradiation time. **(d)** Pseudo-first-order kinetics for AO at pH 7.0 and 10.0 in the presence of the composite nanofibers.

The effect of pH on the photocatalytic degradation of dye was investigated in the range of 7 to 10 at 1E10 − 4 M dye concentration and 0.1 g composite nanofiber loading. Figure 5 shows that the visible light photocatalytic degradation of acridine orange dye increased when increasing the pH 7.0 to 10.0. It is revealed that photocatalytic degradation of dye is a complex process in which physical adsorption of dye on the composite nanofibers is the first step followed by successive decolorization [27]. The photocatalytic performance is ascribed to surface electrical property due to dissimilar interlayer anions. Visible light enhances the charge transformation on the surface of the composite nanofibers and results in hydroxyl radical (·OH) formation which facilitates the degradation. Since pH 10.0 has a significant influence on degradation, it is the recommended pH for the acridine orange dye.

#### Control experiments and photocatalysis reaction

**Figure 6:**
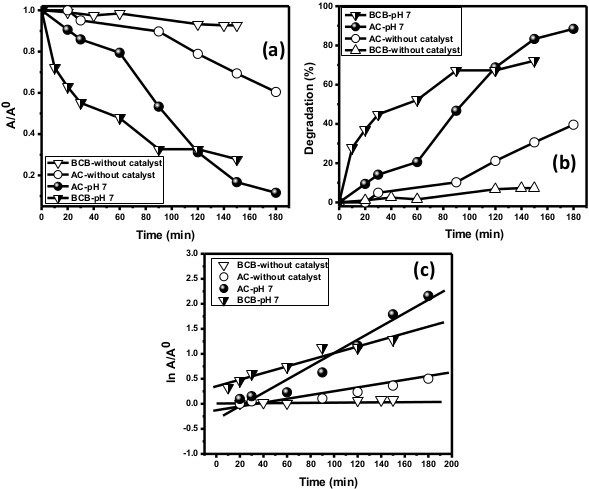
**Composite nanofibers. (a)** Comparison of change in absorbance vs. irradiation time. **(b)** Comparison of percentage of degradation vs. irradiation time. **(c)** Pseudo-first-order kinetics for AO and BCB at pH 7.0 in the presence of the composite nanofibers.

**Figure 7:**
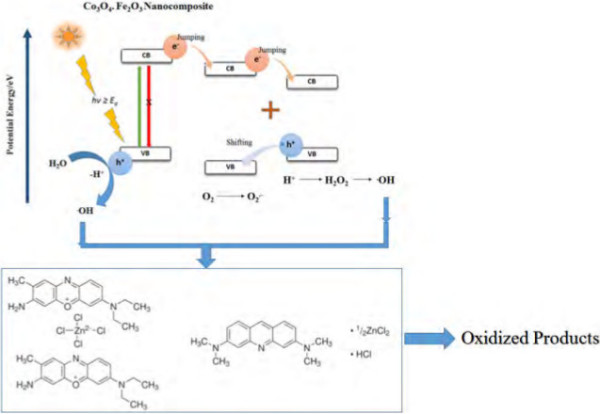
Schematic view of photodegradation using the composite nanofibers.

To evaluate the photocatalytic performance of the composite nanofibers, two different dye solutions (brilliant cresyl blue and acridine orange) were used [2,3]. First, control experiments for both dyes at pH 7.0 were performed without a catalyst under the same visible light conditions. Figure 6 reveals a small fraction of degradation of dye, which defines the process called photolysis reaction (Figure 7). Second, two experiments for AO were performed at pH 7.0 and 10.0 in the presence of the composite nanofibers under solar light irradiation. The composite nanofibers showed best activities towards both dyes (Figure 6). Moreover, Figure 5 shows an excellent absorption spectrum for AO at pH 7.0 and 10.0 which reflected about 97% decolorization within 3 h.

#### Statistical analysis for the degradation process of dyes

Afterwards, in view of regression analysis, the graph of ln *A*/*A*° vs. time was plotted for the AO dye at pH 7.0 and 10.0 while for the BCB dye at pH 7.0. Figures 5d and 6c show that the best linear relation exists between ordinates and abscissa for AO and BCB dyes, respectively.

#### Kinetics study of dye

In the monitoring of the linear relationship between rate of photocatalytic degradation and initial concentration of reactant, i.e., dye, rate expression 1 represented the best linear relationship which successively followed the Langmuir-Hinshelwood (L-H) kinetics model [27,28]:

(5) $$r=\;-\mathrm{dC}/\mathrm{dt}\;=\;K_{r}\mathrm{KC}\;=\;K_{\mathrm{app}}C$$

where *r* is the degradation rate of dyes (organic pollutant), *K*_*r*_ is the reaction rate constant, *K* is the equilibrium constant, and *C* is the reactant concentration. When *C* was very small, then *KC* was insignificant, so Equation 5 became the first-order kinetics. Under starting conditions of the photocatalytic method (*t* = 0, *C = C°*), Equation 5 was applied to Equation 6:

(6) $$\ln\left(C/C\right)=K_{\mathrm{app}}t$$

where *C°* is the initial concentration of dyes and *C* is the concentration at time *t*. By utilizing Equation 6, we determined the apparent rate constant from the slope of the graph of ln(*C*/*C°*), which corresponds to ln (*A*/*A°*) vs. irradiation interval.

The apparent rate constant of photocatalytic activity showed no dependence on adsorption as well as concentration of the dye present in the solution. Figure 6c illustrates that the degradation of BCB and AO dyes obeyed the first-order kinetics as a plot of the variations of ln (*C*/*C°*) as a function of irradiation time which showed linearity. The corresponding *t*_1/2_ parameters (time required to degrade half of the initial concentration of the dye) and regression relative coefficient values that can easily be calculated are specified in Tables 1 and 2.

**Table 1:** **Pseudo-first-order rate constant and *t***_**1/2 **_**for AO and BCB in the presence of the composite nanofibers**

**Dye**	**pH**	***K***_**app **_**(min**^**−1**^**)**	**Rate of decolorization**	***R***	***R***_**2**_	***t***_**1/2 **_**(min)**	***t***_**1/2 **_**(years)**
AO	7.0	0.0150	1.5000E − 06	0.9928	0.9857	46.1107	0.00009
	10.0	0.0176	1.7700E − 06	0.9715	0.9439	39.2558	0.00007
BCB	7.0	0.0067	0.6720E − 06	0.9720	0.9448	103.1198	0.00020

**Table 2:** **Pseudo-first-order rate constant and *t***_**1/2 **_**for AO and BCB in the absence of the composite nanofibers**

**Dye**	**pH**	***K***_**app **_**(min**^**−1**^**)**	**Rate of decolorization**	***R***	***R***_**2**_	***t***_**1/2 **_**(min)**	***t***_**1/2 **_**(years)**
AO	5.0	0.0027	0.2710E − 06	0.9643	0.9299	255.6364	0.00049
BCB	5.0	0.0006	0.0552E − 06	0.9681	0.9372	1254.3642	0.00239

#### Proposed degradation mechanism

The basic mechanism of the degradation process is depending upon electron-hole (e-h) charge separation. Irradiation of visible light that corresponds to the band gap energy of the composite nanofibers leads the valence band electron to be excited to the conduction band while creating a hole in the valence band. This e*-*h charge separation initiates the radical-generated oxidation-reduction reaction of organic dyes.

These photogenerated electrons strike the surrounding oxygen and convert them into superoxide anion radicals (O_2_^·−^), which are further transformed into H_2_O_2_, followed by · OH radicals after reacting with H^+^ from water molecules. Similarly, the photogenerated hole in the valence band initializes the homolysis of water molecules and introduces · OH radicals which degrade the dye molecules.

Moreover, BCB dye molecules adsorbed on the nanomaterial may be prompted to an excited state in the presence of visible light irradiation. Afterwards, the photoexcited dye inoculated electrons into the conduction band of the nanomaterial via the photosensitization method. On the basis of this discussion, the proposed mechanisms in expression form are as follows:

(7) $${\mathrm{Co}}_{3}O_{4}\cdot {\mathrm{Fe}}_{2}O_{3}+\mathrm{hv}\to {\mathrm{Co}}_{3}O_{4}\cdot {\mathrm{Fe}}_{2}O_{3}\left({e_{\mathrm{CB}}}^{-}+{h_{\mathrm{VB}}}^{+}\right)$$

(8) $${\mathrm{Co}}_{3}O_{4}\cdot {\mathrm{Fe}}_{2}O_{3}\left({e_{\mathrm{CB}}}^{-}\right)\;+\;O_{2}\to {\mathrm{Co}}_{3}O_{4}\cdot {\mathrm{Fe}}_{2}O_{3}{{+\;O}_{2}}^{\cdot -}$$

(9) $${O_{2}}^{\cdot -}+\;H^{+}\left(H_{2}O\right)\to H_{2}O_{2}+\cdot \mathrm{OH}$$

(10) $${\mathrm{Co}}_{3}O_{4}\cdot {\mathrm{Fe}}_{2}O_{3}\left({h_{\mathrm{VB}}}^{+}\right)+H_{2}O\to {\mathrm{Co}}_{3}O_{4}\cdot {\mathrm{Fe}}_{2}O_{3}+\cdot \mathrm{OH}$$

(11) ⋅OH+AO/BCB→Oxidizedproducts

## Conclusions

Well-crystalline Co_3_O_4_/Fe_2_O_3_ composite nanofibers were prepared by a low-temperature process and examined by different spectroscopic techniques. The nanofibers were optically active and showed potential application as a solar photocatalyst by well-organized degradation of AO and BCB. Thus, it is concluded that nanofibers are an active photocatalyst for achieving capable photocatalysts in favor of water resources and health observation.

## Competing interests

The authors declare that they have no competing interests.

## Authors' contributions

SBK and AMA prepared the nanomaterials, carried out the structural analyses of the samples, scrutinized the experimental outcomes, and took part in the manuscript preparation. SABA organized the study, studied the data, carried out the degradation work, and contributed to the manuscript writing. All authors read and approved the final manuscript.
